# The association between orthostatic hypotension and cognitive state among adults 65 years and older who underwent a comprehensive geriatric assessment

**DOI:** 10.1097/MD.0000000000004264

**Published:** 2016-07-22

**Authors:** Boris Punchick, Tamar Freud, Yan Press

**Affiliations:** a Yasski Clinic, Comprehensive Geriatric Assessment Unit, Clalit Health Services, Beer-Sheva, Israel; b Unit for Community Geriatrics, Division of Health in the Community, Ben-Gurion University of the Negev, Beer-Sheva, Israel; c Department of Family Medicine, Siaal Research Center for Family Medicine and Primary Care, Faculty of Health Sciences, Ben-Gurion University of the Negev, Beer-Sheva, Israel.

**Keywords:** cognitive impairment, elderly, geriatric assessment, orthostatic hypotension

## Abstract

The prevalence of cognitive impairment and orthostatic hypotension (OH) increases with age, but the results of studies that assessed possible associations between them are inconsistent.

The aim of this study is to assess possible associations between cognitive impairment and OH in patients ≥65 years of age who underwent a comprehensive geriatric assessment.

A retrospective analysis was conducted of the computerized medical records of the study population from 2005 to 2013. Data collected included blood pressure measurements that enabled the calculation of OH, results of the mini-mental state examination (MMSE), results of the Montreal cognitive assessment (MoCA) test, and cognitive diagnoses that were determined over the course of the assessment.

The rate of OH in the study population of 571 adults was 32.1%. The mean MMSE score was 22.5 ± 5.2 among participants with OH and 21.6 ± 5.8 among those without OH (*P* = 0.09). The absence of a significant association between OH and MMSE remained after adjusting the MMSE score for age and education level. The mean MoCA score was 16.4 ± 5.0 among participants with OH and 16.4 ± 4.8 among those without (*P* = 0.33). The prevalence of OH was 39% among participants without cognitive impairment, 28.9% among those with mild cognitive impairment (MCI), and 30.6% among those with dementia (*P* = 0.13).

There was no association between OH and cognitive impairment in adults who underwent a comprehensive geriatric assessment.

## Abstract 2

Supplemental Digital Content is available in the text

## 1 Introduction

Orthostatic hypotension (OH) is a common entity among elderly patients with a prevalence that ranges from 5.8% in community-dwelling elderly^[1]^ to over 50% in selected populations.^[2,3]^

Cognitive impairment is also common in the population of adults ≥65 years. It is estimated that ∼44 million people in the world suffer from dementia.^[4]^

There are several potential mechanisms for an association between OH and cognitive impairment. Postural dysregulation of blood pressure can be an indicator of functional as opposed to chronological age and significant, persistent changes in blood pressure can have a negative effect on brain perfusion causing changes such as leukoaraiosis that can damage cognitive function.^[5]^ The results of a study in rats showed that brain perfusion enhances amyloid precursor protein mRNA expression.^[6]^

The association between OH and cognitive impairment was also evaluated in several clinical studies. In some^[5,7–15]^ an association was found, whereas in others^[1,2,6,16–28]^ no association was seen.

In the present study, we conducted data analyses on a research database to evaluate the rate of OH in patients ≥65 years of age who came for a comprehensive geriatric assessment.^[29]^

## 2 Methods

### 2.1 Study population

The study was conducted among male and female patients, ≥65 years of age, and who came for a comprehensive geriatric assessment. The details of the study have been published previously.^[29]^

In short, information about the patients who were referred by their family doctor for a geriatric assessment and were evaluated over the years 2005 to 2013 was obtained from the computerized medical records of the of the Outpatient Unit for Comprehensive Geriatric Assessment. The data included sociodemographic information, comorbidity, and an extensive physical examination that included blood pressure measurements conducted to enable calculation of OH. Over the course of the geriatric assessment the patients underwent a cognitive evaluation that included, in most cases, the mini-mental state examination (MMSE)^[31]^ and, in some cases, the Montreal cognitive assessment test (MoCA).^[30]^ The Helsinki Committee of the Meir Medical Center approved the study (Approval #72/2011 [k]).

### 2.2 Blood pressure measurements and definitions of OH

Blood pressure measurements were made with an electronic instrument (Automatic Scholar III EL Monitor, Criticare Systems, Inc, Waukesha, WI) between the hours of 8 AM and 4 PM. The first measurement was taken after the patient laid in the supine position in a quiet room for 10 minutes. Blood pressure was measured again during the first and third minutes after standing up.

For the present study, OH was defined as a drop in systolic blood pressure of ≥20 mm Hg and/or a drop in diastolic blood pressure of ≥10 mm Hg at the 1st and/or the 3rd minute measurements upright compared with the measurement at the 10th minute supine.

### 2.3 Definitions of cognitive impairment

In the present study the cognitive state was defined by the following:

1. MMSE score
2. Delta MMSE score—the difference between the MMSE score from the geriatric assessment and patient's expected MMSE by age and education level according to the Crum table.^[31]^ If the study MMSE score was equal to or above the expected score by age and education level the patient was categorized as “normal delta MMSE.” If the study MMSE score was lower than the expected MMSE score it was categorized as “abnormal delta MMSE.” Since data on educational level were not available in all the patient records, the delta MMSE could only be calculated for the majority of the study patients, not all of them.
3. MoCA score—this test was only conducted if there was a clinical indication, so data were only available for some of the participants.
4. Dementia and mild cognitive impairment (MCI)—these diagnoses were determined during the geriatric assessment. MCI was diagnosed if there was a cognitive decline that did not affect the patient's function.^[32]^ Dementia was diagnosed in accordance with the DSM-IV criteria.^[33]^ In some of the cases, where the diagnosis was listed as “suspected dementia,” the patients were categorized as dementia for the purposes of the study. In the majority of cases in which the diagnosis was dementia there was no information as to the type of dementia so the data analyses could not be performed on sub-groups by dementia type.

### 2.4 Statistical analysis

Results of continuous variables are reported as mean ± SD. Results of categorical variables are described as frequencies. Continuous variables were compared using the Student *t* test and analysis of variance, and categorical variables using the χ^2^ test. Univariate analysis was performed in order to identify variables that might be related to cognitive state. Statistical analysis was performed using the SPSS software, version 22 (Chicago, IL). We had intended to construct a multivariate model with the aim of taking into account all confounders. However, as in the univariate analyses there were no statistically significant differences in cognitive state between patients with and without OH, a multivariable analysis was not conducted.

## 3 Results

Over the course of the study period a total of 628 patients were examined in the Geriatric Assessment Unit. Of these, 571 (90.9%) had blood pressure (BP) measurements that enabled calculation of OH, as described in a previous paper.^[29]^ The prevalence of OH was 32.1%. There were MMSE scores for 534 patients (85 % of the entire study population of 571 patients): 172 with OH (mean MMSE score = 22.5 ± 5.2) and 362 without OH (mean MMSE score = 21.6 ± 5.8) (*P* = 0.09). The MoCA test was conducted on 258 patients (45.2% of the entire study population of 571 patients), 87 with OH (mean MoCA score = 16.4 ± 5.0) and 171 without OH (mean score = 16.4 ± 4.8) (*P* = 0.33).

Table 1 shows the results of the cognitive assessment among participants with and without OH. There were no differences between the groups in diagnoses related to cognitive impairment including delta MMSE and MoCA score.

**Table 1:**
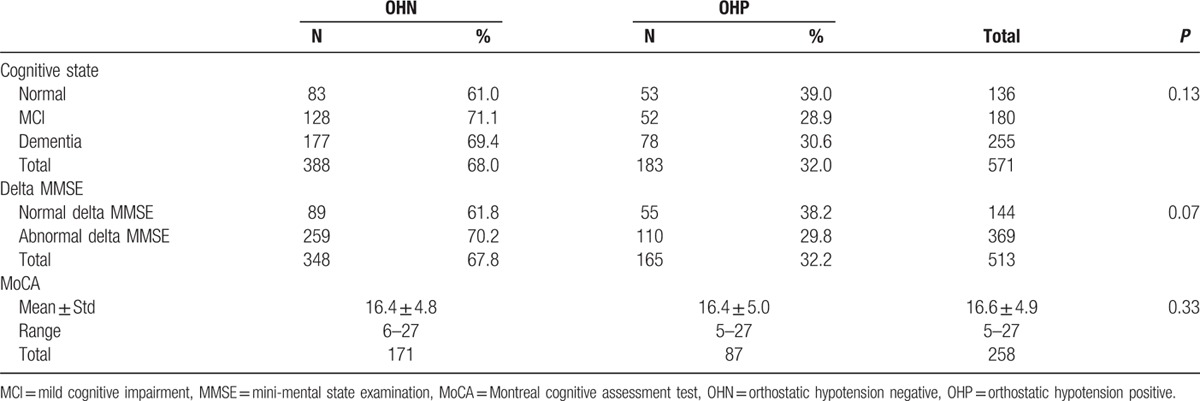
Cognitive state in patients with and without orthostatic hypotension.

Two hundred ninety-four of the 571 patients included in the study (51.5%) had blood pressure measurements ≥140 mm Hg. One study reported an association between OH and cognitive state only if the OH was related to high supine blood pressure^[34]^ and another study reported an association between OH and cognitive state if baseline blood pressure was low.^[28]^ In light of these reports we performed an additional analysis of delta MMSE and diagnoses of cognitive impairment with the patients assigned to one group with supine systolic BP < 140 mm Hg and a second group with supine systolic BP ≥140 mm Hg. This new analysis did not change the results since again there was no association between OH and delta MMSE or cognitive diagnoses in either group (Table 2).

**Table 2:**
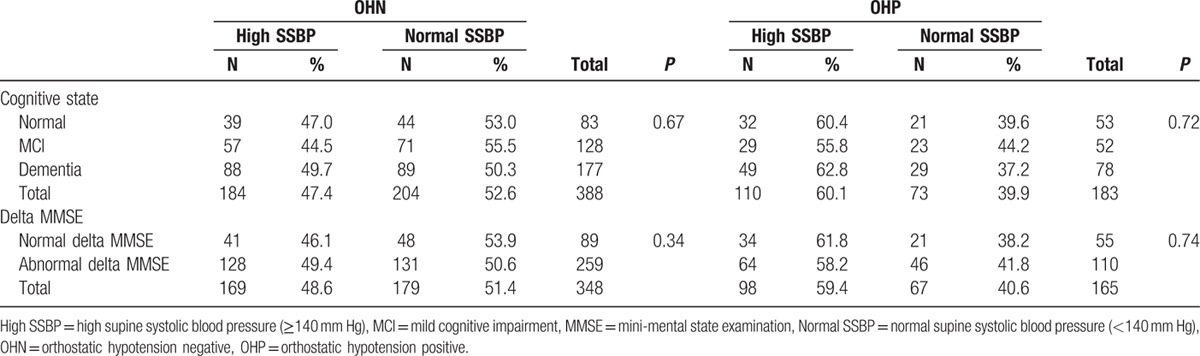
The association between cognitive state and orthostatic hypotension in patients with normal and high supine systolic blood pressure.

Since no significant association was found in the univariate analyses between OH and cognitive state, there is no reason to construct a multivariate model to assess the effect of other factors, such as comorbidity or drugs that could affect the cognitive state or cause OH. The comorbidity rate and drug therapy of the study population was described in our previous study.^[35]^

## 4 Discussion

In the present study of patients who underwent a comprehensive geriatric assessment there was no association between cognitive state and OH. These results are consistent with the results of some of the previously reported studies and in disagreement with the conclusions of other studies (see supplement Table 1—Summary of studies evaluating associations between Orthostatic Hypotension and cognitive state in the elderly). An association has been reported between OH and cognitive impairment in studies on Parkinson patients^[7,10,15]^ or in settings with a very high rate of cognitive impairment.^[9,11,13,16]^ Nevertheless, an association between OH and cognitive impairment was also found in studies that evaluated nonselective adult populations.^[5,10]^

In the Irish Longitudinal Study on Aging (TILDA), with a study population 4690 participants, Frewen et al^[39]^ found an association between OH and cognitive impairment only among those OH patients who also had supine hypertension. In contrast, there are many studies in which no association was found between OH and cognitive changes, including community-based studies^[1,18,21,24,27,28]^ and studies in assisted living homes,^[21,23,24,27]^ among hospitalized patients^[16,17,36]^ and among patients in fall clinics.^[2,19,25]^ The present study is more similar, in terms of the composition of the study population (age and co-morbidity) and the prevalence of OH, to studies that were conducted in selected populations of elderly patients with a heavy burden of comorbidity, such as residents in assisted living homes, hospitalized elderly patients, and patients in fall clinics. These similarities may explain why no association was found in the present study between OH and cognitive state.

The difference in results in the various studies could stem from the spectrum of causes of chronic autonomic failure with symptoms of OH in different populations such as primary (Parkinson disease, Lewy body dementia, multiple system atrophy, etc.) or secondary (drug-related OH, diabetes mellitus, cardiovascular diseases, etc.). In one third of the cases the etiology of OH is not clear.^[37]^

In the present study, MMSE was used as one of the measures of cognitive state. MMSE was used in three studies in which an association was found between OH and cognitive state, based on a neuropsychological assessment.^[5,8,9]^ In two of these studies,^[5,10]^ there was no difference in the MMSE score between participants with and without OH. MMSE was also used in 8 studies in which no association was found between OH and cognitive impairment.^[1,18,22,25–28,36]^ In only one of those studies,^[28]^ the prospective Singapore Longitudinal Aging Study with 2292 participants, was there a statistically significant difference in MMSE score, but after adjusting for relevant variables OH was no longer associated with cognitive state at baseline or with cognitive decline on follow-up. Thus, one can assume that when there is a difference in cognitive state between groups of individuals with and without OH, the difference is not strong enough to be reflected in the MMSE scores. In the present study we also used MoCA, but this measure was missing in half of the participants so that its contribution to the understanding of a possible association between OH and cognitive state in the present study is limited. In any event, OH rates in the groups of patients with dementia, MCI or no cognitive impairment in the present study do not support the existence of an association between OH and a decline in cognitive state.

### 4.1 Strengths and limitations

The strength of the present study is the relatively large number of participants from a generally homogeneously population of elderly frail. An additional strength is that throughout the study years only one nurse conducted all blood pressure measurements, thus reducing the risk of technical inaccuracy in the measurements.

Our study has several limitations. First, it is a cross-sectional, retrospective study. In this study design it is difficult to account for all of the potential confounders. Furthermore, in this type of study, even when most confounders are successfully taken into account, one can only reach conclusions as to possible associations between cognitive impairment and OH, but not about a causal relationship between OH and the future development of cognitive impairment.

Second, blood pressure was measured only once over the course of the study. As there are natural oscillations in blood pressure throughout the day,^[38–41]^ the absence of an association between OH and cognitive state in our study could be coincidental. Third, as the study represents a retrospective description of the daily practice of a unit whose main aim is treatment, not research, there could be a degree of bias in diagnoses and conclusions even though the unit medical staff applied accepted diagnostic criteria conscientiously.

As this is a descriptive study of a daily clinical practice and not a pre-designed study, there was no selection process for the study sample. The medical charts contained MMSE scores for only 534 of the 628 patients who were tested during the study period (85.0%) and MoCA scores for only 258 of the 628 (41.1%). This could lead to a possible bias in relation to the conclusions of the study.

Another significant limitation is that because of the nature of the study it was not possible to get data on dementia subtype, so we could not conduct sub-analyses on this variable. Of course, the lack of association between OH and cognitive state during the assessment session does not totally negate the possibility that cognitive impairment is present at a higher rate among OH patients.

In conclusion, in a study group of patients who underwent a comprehensive geriatric assessment no association was found between OH and cognitive impairment. A prospective study should be conducted among the frail elderly including a comprehensive neuropsychological assessment.

## Supplementary Material

Supplemental Digital Content
