# Supplementary material for: The association between orthostatic hypotension and cognitive state among adults 65 years and older who underwent a comprehensive geriatric assessment
Source: Medicine (Baltimore). 2016 Jul 22;95(29):e4264. doi: 10.1097/MD.0000000000004264 (PMC5265775; doi:10.1097/MD.0000000000004264)
Supplement: Supplemental Digital Content [file medi-95-e4264-s001.doc]

**Supplement table 1**: Summary of studies that evaluated associations between Orthostatic Hypotension and cognitive state in the elderly

| **Reference** | | **Type of study** | | **Type/study population** | | **Age** | | **Overall OHP** | | **MMSE score** | | | | | | **Study conclusions** |
| --- | --- | --- | --- | --- | --- | --- | --- | --- | --- | --- | --- | --- | --- | --- | --- | --- |
|  |  |  |  |  |  |  |  |  |  | **OHP** | | **OHN** | | **P** | |  |
|  |  |  |  |  |  |  |  | **(%)** | | **mean±SD** | | **mean±SD** | |  |  |  |
| Allan et al., 2007 ^7^ | | Cross-sectional | | Selected: 177 patients with dementia from psychiatric, neurologic and geriatric clinics and 38 controls | | >72 | | 13-52 | | - | | - | | - | | The rate of sustained OH among patients with dementia was higher (34%-52%) than in the control group (13%) |
| Allcock et al.,2006 ^8^ | | Cross-sectional | | Selected:175 patients with PD | | OHP-72.4 OHN-69.2 | | 49.7 | | 25.0±4.0 | | 26.0±5.0 | | 0.14 | | An MMSE score below 24 was found in 33% in the OHP group compared to 27% in the OHN group (NS). The scores for sustained attention and visual memory tests were lower in the OH group. |
| LEA et al., 2011 ^22^ | | Cross-sectional | | Selected:133 patients in LTC | | 82.4 ± 9.5 | | 29.3 | | 15.5 ± 7.2 | | 16.1 ± 7.9 | | 0.6 | | In the OHP group 2.6% had AD and 25.6% had VaD, compared to 2.1% and 23.4%, respectively in the OHN group (NS). |
| Aung et al., 2012 ^16^ | | Cross-sectional | | Selected:76 inpatients from general, acute, trauma and surgical wards | | 67.8 ±19.6 | | 23.7 | | - | | - | | - | | In the OHP group 5.6% had dementia compared to 6.9% in the OHN group (NS) |
| Bengtsson-Lindberg et at., 2014 ^9^ | | Cross-sectional | | Selected: 204 patients, 154 demented patients and 50 controls from the Malmo Alzheimer study | | 76±6 | | 43 | | 22.5±5.1 | | 24.8 ±4.8 | | p<0.001 | | The rate of OH was 53% in the dementia group compared to 14% in the control group. |
| Boddaert, Tamim et al., 2004^17^ | | Cross-sectional | | Selected: 57 patients admitted to a geriatrics ward with a history of recent falls. | | 84.2 ± 6.7 | | 31.6 | | - | | - | | - | | In the OHP group 22% had dementia compared to 31% in the OHN group (NS). |
| Cooke et al., 2013^18^ | | Cross-sectional | | Non-selected: 326 participants from the Health Inequalities and Ageing in the Community Evaluation study | | 65+ | | 58.6 | | 30 | | 30 | | 0.6 | | No difference in MMSE score between the OHP and OHN groups. |
| Elmstahl &Widerstrom, 2014^10^ | | Prospective | | Non-selected: 2,931 participants in the Good Aging in Skane Study | | 68.0±8.5 | | 18 | | - | | - | | - | | Patients with OHP at baseline had a higher risk (OR=1.9) to develop dementia after 6 years than patients with OHN. There was no statistically significant difference in the risk for MCI. |
| Frewen et al., 2014^34^ | | Cross-sectional | | Non-selected: 4,690 participants in The Irish Longitudinal Study on Ageing | | 60.9±5.8 | | 20 seconds after standing up-29.2; after 30 seconds-15; after 60 seconds-6.6; after 90 seconds-4.6. | | - | | - | | - | | The rates of global executive function, sustained attention memory and processing speed impairment were not higher in the OHP group. However, in the OHP group with SH the global executive function was impaired. The authors concluded that the association between OH and SH causes cognitive impairment. |
| Gangavati et al., 2011 ^1^ | | Prospective (cross-sectional analysis) | | Non-selected: 722 community-dwelling elderly from the MOBILIZE Boston Study | | 78.5 | | 5.8 | | 27.1 ± 2.6 | | 27.1 ± 2.7 | | 0.96 | | There was no difference in MMSE between the two study groups. |
| Gaxatte et al., 2013 ^19^ | | Cross-sectional | | Selected: 833 elderly fallers | | 80.4 ±7.4 | | 23.9 | | - | | - | | - | | In the OHP group 46% had dementia compared to 42% in the OHN group (NS). |
| Hartog et al., 2015 ^20^ | | Prospective cohort (cross-sectional analysis) | | Selected:290 patients from a nursing home facility; 106 from a psychogeriatric department, 56 from a somatic department, and 128 from a rehab department | | 80.9±9.9 | | 36.6 | | - | | - | | - | | Dementia did not increase the risk for OH (OR=1.18; 95% CI 0.73-1.92) |
| Hiitola et al., 2009 ^21^ | | Cross-sectional | | Non-selected: 653 participants in the Geriatric Multidisciplinary Strategy for the  Good Care of the Elderly study | | ≥ 75 | | 34 | | - | | - | | - | | The rate of dementia was 17% in the OHP group compared to 12% in the OHN group (NS). |
| Largo et al., 2012 ^2^ | | Cross-sectional | | Selected: 313 patients from an outpatient fall clinic | | > 77.3 | | 54 | | - | | - | | - | | The rate of dementia was 8% in the OHP group compared to 7% in the OHN group (NS). |
| Matsubayashi et al.,1997 ^5^ | Cross-sectional | | Non-selected: 334 patients from the Kahoku Study | | 80±5 | | 6 | | 25.8 ±3.8 | | 26.5±3.4 | | NS | | There was no difference between the HP and OHN groups in MMSE, but the OHP group had worse scores in visual memory and visuospatial performance. | |
| Mehrabian et al., 2010 ^11^ | Cross-sectional | | Selected: 495 patients from a memory clinic | | 76±8 | | 14 | | - | | - | | - | | The rate of OH was higher in dementia patients (15% in AD and 22% in VaD) and in MCI (12%) than in patients with no cognitive problem (4%, P<0.01). In addition, the Cognitive Efficiency Profile score was higher (24±50 vs. 22±56 (P<0.05). | |
| Ooi et al., 1997 ^23^ | Cross-sectional | | Selected: 911 elderly participants from nursing homes | | ≥60; at least 72% ≥ 85 | | 68.3 | | - | | - | | - | | The rate of patients with dementia was 27.2%-33.0% in the OHP groups compared to 27.6% in the OHN group (NS). | |
| Passant et al., 1997 ^12^ | Cross-sectional | | Selected: 151 demented patients from a psychogeriatric department | | from 40 to 94; | | 46 had OH or supine SBP <100 mm Hg | | - | | - | | - | | The rate of OH or supine hypotension was 46% in the group of patients with dementia (there was no control group). | |
| Pilleri et al., 2012 ^14^ | Cross-sectional | | Selected: 48 patients with PD | | 65 | | 48 | | - | | - | | - | | The MMSE score was below 24 in 34.7% of the OHP group and 32.0% of the OHN group (NS). There was a statistically significant difference in verbal memory, and visuospatial attention with higher scores in the OHN group. | |
| Rose et al., 2010 ^24^ | Prospective | | Non-selected: 12,702 participants in the Atherosclerosis Risk in Communities Study | | > 53 | | 5.1 | | - | | - | | - | | After adjusting for age and other cardiovascular risk factors there was no association between OH and the cognitive functions that were assessed. | |
| Schoon et al., 2012 ^25^ | Cross-sectional | | Selected: 178 participants from a falls and syncope clinic | | 80.2+6.8 | | NA | | 24.9 ±4.6 | | 25.7 ±3.9 | | 0.27 | | The rate of cognitive impairment was 43% in the OHP group and 39% in the OHN group (NS). | |
| Shen et al., 2015 ^36^ | Cross-sectional | | Selected: 176 patients with HTN from a geriatric ward | | 76.7±6.6 | | 20.5 | | median 27 | | median 27 | | 0.93 | | The MMSE score was identical in the OHP and OHN groups. | |
| Sonnesyn et al., 2009 ^15^ | Cross-sectional | | Selected: 262 participants: 158 patients with mild dementia and 104 controls | | ≥ 70 | | 41 | | - | | - | | - | | OH was found in 41% of dementia patients compared to 14% of the controls (P=0.000). | |
| Valbusa et al., 2011^26^ | Cross-sectional | | Selected: 994 participants in the PARTAGE study | | 80+, mean 88±5 | | 18 | | 23±5 | | 23±5 | | NS | | The rate of dementia was 17% in the OHP group and 17% in the OHN group. | |
| Viramo et al., 1999^27^ | Prospective | | Non-selected: 641 individuals in five rural municipalities around the town of Oulu in Northern Finland | | 76 | | 28.7 | | 21.6±4.0 | | 21.2±4.1 | | NS | | There was no difference in the change in MMSE between the OHP and OHN groups after a follow-up of 2.5 years. | |
| Yap et al., 2008^28^ | Prospective | | Non-selected: 2,292 participants in the Singapore Longitudinal Aging Studies | | 65.5±7.4 | | 16.6 | | 26.7±3.5 | | 27.3±3.2 | | 0.003 | | Although the MMSE score was lower in the OHP group at baseline, after adjusting for age and other relevant variables there was no association with either cognitive impairment at baseline or cognitive decline at follow-up. | |

| **OHP**- orthostatic hypotension positive | **DLB** - Dementia of Lewy Bodies |  |
| --- | --- | --- |
| **OHN**- Orthostatic hypotension negative | **PDD**- Parkinson's Disease Dementia |  |
| **PD**- Parkinson's Disease | **LTC**- Long- Term Care |  |
| **AD**- Alzheimer's Disease | **HTN**- hypertension |  |
| **VaD**- Vascular Dementia | **SH**- Supine Hypertension |  |
